# Long term adherence to continuous positive Airway pressure in mild obstructive sleep apnea

**DOI:** 10.1186/s12890-023-02612-3

**Published:** 2023-09-01

**Authors:** Min Qiao, Yiyu Xie, Armand Wolff, Jeff Kwon

**Affiliations:** 1 Pulmonary and critical care medicine, University of Rochester Medical Center, 601 Elmwood Ave, 14642 Rochester, NY USA; 2 Medicine department, University of Massachusetts Chan Medical School, 55 Lake Ave, North Worcester, 01655 Worcester, MA USA; 3 Pulmonary disease, critical care and sleep medicine, 267 Grant St, Yale New Haven Health Bridgeport Hospital, Bridgeport, CT 06610 USA

**Keywords:** Mild sleep apnea, CPAP compliance, CPAP adherence, Home sleep apnea testing

## Abstract

**Background:**

Studies have shown that a significant percentage of patients with obstructive sleep apnea (OSA) do not tolerate continuous positive airway pressure (CPAP) therapy and long-term use may be as low as 30%. Given the lower levels of symptoms and health-related risks, patients with mild sleep apnea may be at even higher risk for non-adherence to long term CPAP. The purpose of our study was to investigate the prevalence and associations of long-term CPAP adherence in first time users with mild sleep apnea diagnosed by home sleep apnea testing (HSAT).

**Methods:**

We identified all the patients who were diagnosed with mild sleep apnea (5 = < AHI < 15) by home sleep apnea testing from 01/2013 to 06/2019 at a large, combined community and hospital-based sleep practice. Only first time CPAP users were included. Compliance was defined as CPAP usage ≥ 4 h per night on ≥ 70% of nights over 30 consecutive days. We defined long term adherence as compliance on the 12th month following CPAP set up. Patient demographics, comorbidities, and CPAP compliance at 1st, 3rd, 6th, 9th and 12th month after therapy initiation were collected. We compared and identified the factors that had significant difference (P < 0.1) between compliant and non-compliant groups at the 12th month.

**Results:**

222 patients were included in the analysis. 57 (25.7%) patients were adherent with long term CPAP treatment. The following factors were associated with a greater likelihood for long-term CPAP adherence: older age, lower body mass index (BMI), presence of a bed partner, non-smoker, presence of Diabetes Mellitus (DM), presence of Heart Failure (CHF), lack of depression, and compliance at 1st, 3rd, 6th and 9th month.

**Conclusions:**

Long term CPAP compliance in mild sleep apnea patients is low. Long term adherence to CPAP can be predicted based on CPAP adherence during the first three months.

**Supplementary Information:**

The online version contains supplementary material available at 10.1186/s12890-023-02612-3.

## Background

Mild obstructive sleep apnea (OSA) is defined as an apnea-hypopnea index of 5–15 events/hour. It is a highly prevalent disorder and is estimated to account for 50 to 70% of all cases of OSA [1]. Due to the widely publicized associations between OSA and adverse cardio-metabolic health outcomes, it is common practice to offer treatment with continuous positive airway pressure (CPAP), even to those who have mild OSA [2, 3]. According to the American Academy of Sleep Medicine practice guidelines, CPAP treatment for mild OSA is considered optional for symptom management or for patients with significant co-morbidities [4].

Intolerance of CPAP therapy is a common challenge encountered in clinical practice. Observational studies suggest adherence(compliance) with CPAP therapy is between 30 to 60% [5]. In mild OSA, in which treatable symptoms are often less severe, adherence to CPAP therapy may be even lower [6]. Reasons for poor adherence include a variety of medical and non-medical factors, such as physical discomfort from wearing the CPAP apparatus, financial burden, and psychological stress [6].

Over the past 15 years, the practice of sleep medicine has changed significantly with the widespread integration of home-based sleep testing and treatment. However, the impact of home-based testing on the long term acceptance of CPAP treatment is not well known. This is particularly true patients with mild OSA, a group that may be at higher risk for poor adherence to long term treatment. The purpose of our study is to investigate the prevalence of long-term CPAP adherence in first time users with mild OSA diagnosed by home sleep apnea testing (HSAT) and to identify the factors associated with long-term CPAP use.

## Methods

### Study population and design

We conducted a retrospective observational study at the sleep center of a 500-bed University affiliated community teaching hospital in Connecticut that has a diverse patient population. All patients included in this study underwent evaluation by one of the three sleep specialists prior to a home sleep study. Patients were evaluated at either a community-based office by a private sleep medicine specialist (T) or at a hospital-based clinic by one of the two pulmonary and sleep medicine attending physicians (K or W). Patients diagnosed with mild OSA were provided with regular routine office follow up with their treating sleep physician to assess adherence and effectiveness of CPAP therapy. Patients with poor CPAP compliance were routinely assessed for reasons for treatment intolerance, such as claustrophobia, pressure intolerance, skin irritation caused by the mask, inappropriate CPAP pressure settings, and complications of nasal disease in follow up visits. All the above-mentioned conditions were assessed and addressed accordingly at the discretion of the treating sleep physician.

To protect patient confidentiality, each patient was assigned a code number and no personal identifying information was included in the data set. The study was approved by the Yale New Haven Health-Bridgeport Hospital Institutional Review Board (IRB#082010).

### Data source

We identified all patients 18 years and older diagnosed with mild obstructive sleep apnea (5 ≤ AHI < 15) by HSAT using a type 3 portable monitor (Nox-T3, Nox Medical Inc., Reykjavik, Iceland) between 01/2013 and 06/2019. This device measures airflow by nasal pressure transduction, respiratory effort using inductance plethysmography, snoring, body position, activity (movement), and oxygen saturation by pulse oximetry. Analysis start time and stop time on the HSAT recordings were determined based on a standard post-study questionnaire and the activity signal on the recording. All studies were initially scored automatically using Noxturnal software and then reviewed and re-scored as necessary by the consulting provider. Using American Academy of Sleep Medicine 2012 scoring criteria, an obstructive apnea was defined as a ≥ 90% reduction in airflow from baseline for at least 10 s associated with ongoing respiratory effort. A hypopnea was defined as a ≥ 30% reduction in the airflow signal for ≥ 10 s associated with either a ≥ 3% or 4% reduction in oxygen saturation, depending on insurance coverage [7]. The average number of apneas and hypopneas per hour (apnea-hypopnea index, AHI) was determined based on the total analysis time of the HSAT recordings.

We identified patients with mild OSA and collected patient demographics (age, gender, race and ethnicity, body mass index (BMI), bed partner status, smoking history) and data regarding relevant comorbidities (hypertension (HTN), Diabetes Mellitus (DM), stroke, depression, anxiety, coronary artery disease (CAD), congestive heart failure (CHF), pulmonary hypertension (PHT), allergic rhinitis (AR), and atrial fibrillation (AF)). The information about comorbidities were recorded and gathered through review of sleep physician evaluations contained in electronic medical records. Our institution has been using Epic EMR for more than 10 years in both the inpatient and outpatient practices. There were 3 ways we were able collect the information. (1) The sleep center requested all the patients complete a questionnaire on comorbidities, which was scanned into the EMR. (2) Review of the initial consultation notes by the attending physicians which always include current medications and past medical history/comorbidities. Both the questionnaire and the initial consult note were reviewed to gather the information. (3) Epic search was done using the disease name in situations when the comorbidities were not mentioned in the aforementioned sleep center questionnaire or initial consult note.

Epworth Sleepiness Scores (ESS) was assessed before and after CPAP treatment. Data regarding the primary practice setting of the consulting attending physicians, insurance types, and CPAP settings (either auto PAP or fixed pressure through PAP titration) were obtained through electronic medical record review.

CPAP usage, pressure setting, and mask leak data were collected via AirView™ (Resmed) at the 1st, 3rd, 6th, 9th and 12th month after therapy initiation. Non-Resmed CPAP machine users were excluded from the study, as greater than 90% of patients were provided a Resmed CPAP machine during this period.

### Exclusion criteria

All the patients underwent a comprehensive sleep consultation by one of the 3 Sleep Medicine- boarded specialists in their office. Based on their comprehensive evaluations, a variety of sleep disorders were diagnosed by these specialists. Patients who had poorly controlled sleep disorders other than OSA felt to be contributing to symptoms as documented by the treating physician, were excluded from the analysis.

Exclusion criteria also included prior CPAP users, those who were not offered CPAP, who declined it, who were recommended against CPAP by their treating physician, those who agreed to therapy but never initiated CPAP use, those who had initiated CPAP but for whom adherence data were not available, and patients who underwent bariatric surgery within 1 year of their sleep study. We also excluded patients whose HSAT was not valid due to poor quality, were falsely positive (confirmed *not* to have mild sleep apnea on a subsequent sleep study), demonstrated predominant central sleep apnea, or were done to test the efficacy of an oral appliance.

### Adherence (compliance)

Adherence (Compliance) was defined as use of CPAP for a minimum of ≥ 4 h on at least ≥ 70% of nights over 30 consecutive days. Long term adherence was determined at the 12th month following CPAP initiation.

### Groups

Included patients were divided into groups based on compliance at the 12th month.

We identified the variables in which there was significant difference (P < 0.1) between compliance groups at the 12th month.

### Statistical analysis

Chi-squared test, Fisher exact test and t-test were performed to analyze the clinical characteristics and CPAP related variables (Tables 1 and 2). The variates with p value less than 0.1were included in the multivariate logistic regression analyses (Table 3). Using Fisher exact test and the adjusted p-values by false detection rate, we compared different attending physicians and insurance types (Table S1-3). Chi-squared test, Fisher exact test and t-test (Table S4) and propensity scores (Table S5) were also used to further analyze the difference between attending physicians. Statistical software R project 4.0.5 was used.

**Table 1:** Clinical characteristics of the patients between the compliant and non-compliant groups

Clinical Characteristics		Non-compliant at 12th month	Compliant at 12th month	P-value
		N = 165	N = 57	
Age in years	Mean	48.4	52.9	0.004177
	Range	(20,79)	(32,72)	
Gender	Female	96	34	0.8772
	Male	69	23	
Race and Ethnicity	Black	40	12	0.5331
	Asian	2	0	
	Hispanic	65	19	
	White	58	26	
BMI	Mean	35.7	33.6	0.04491
	Range	(21,67)	(21,49)	
CPAP Setting	auto PAP	154	53	1
	Fixed pressure	11	4	
HTN	No	79	22	0.2803
	Yes	86	35	
Depression	No	123	50	0.04222
	Yes	42	7	
Anxiety	No	132	42	0.3521
	Yes	33	15	
Stroke	No	163	57	1
	Yes	2	0	
AF	No	160	53	0.2397
	Yes	5	4	
DM	No	124	36	0.08911
	Yes	41	21	
CAD	No	159	52	0.1551
	Yes	6	5	
CHF	No	164	52	0.004682
	Yes	1	5	
PHT	No	164	55	0.1627
	Yes	1	2	
AR	No	136	48	0.8406
	Yes	29	9	
AHI	Median	9.1	9.5	0.2639
	Range	(5.1,14.9)	(5,14.7)	
Active smoker	No	137	53	0.08011
	Yes	28	4	
Bed Partner	No	67	15	0.05781
	Yes	98	42	
ESS before therapy	Median	10	10	0.6705
	Range	(0,24)	(0,24)	
ESS after therapy	Median	4	5	0.7177
	Range	(1,14)	(0,14)	
Compliance at 1st month	No	134	19	8.24e-11
	Yes	31	38	
Compliance at 3rd month	No	137	14	1.91e-15
	Yes	28	43	
Compliance at 6th month	No	150	15	< 2.2e-16
	Yes	15	42	
Compliance at 9th month	No	156	12	< 2.2e-16
	Yes	9	45	
Attending physicians	T	47	28	0.01769
	K	44	11	
	W	74	18	
Insurance types	V	77	34	0.08585
	C	12	6	
	D	76	17	

**Table 2:** Comparison of PAP therapy related characteristics between compliant and non-compliant groups among actual CPAP users

Characteristics		Non-compliant at 12th month	Compliant at 12th month	P-value
		N = 150	N = 57	
P95	Median	10.0	10.6	0.07749
	Range	(4.6,14.8)	(6,16.6)	
L95	Median	18.5	11.7	0.007315
	Range	(0,120)	(0.4,55.4)	
L95/P95	Median	1.64	1.04	0.001536
	Range	(0,24.5)	(0.044,5.18)	
Pm	Median	7.45	7.7	0.09225
	Range	(0.9,13.3)	(0.7,13)	
Lm	Median	3.55	1.3	0.01123
	Range	(0,120)	(1,25)	
Lm/Pm	Median	0.534	0.146	0.005667
	Range	(0,24.5)	(0,3.38)	
Post AHI	Median	1.5	1.2	0.1088
	Range	(0,54.8)	(0,4.2)	
Delta AHI	Median	6.8	7.7	0.06454
	Range	(-44.6,14.4)	(2.2,14.2)	

**Table 3:** Effect of Potential Factors on the Adherence of CPAP in 12 months

Variable	Multivariate		Multivariate		Multivariate		Multivariate	
	Odds Ratio (95%CI)	P value	Odds Ratio (95%CI)	P value	Odds Ratio (95%CI)	P value	Odds Ratio (95%CI)	P value
Age (Continuous)	1.02 (0.99,1.06)	0.243	1.03 (0,99,1.07)	0.198	1.03 (0.99,1.07)	0.169	1.02 (0.97,1.06)	0.244
BMI (Continuous)	0.95 (0.88,1.01)	0.097	0.94 (0.88,1.01)	0.107	0.95 (0.88,1.01)	0.116	0.94 (0.88,1.01)	0.094
Depression (Present vs. Not present)	0.43 (0.15, 1.22)	0.114	0.5 (0.17, 146)	0.208	0.43 (0.15, 1.27)	0.128	0.45 (0.16, 1.27)	0.13
DM2 (Present vs. Not present)	2.44 (1.01, 5.87)	0.046	3.18 (1.25, 8.13)	0.016	3.64 (1.38, 9.56)	0.009	2.38 (0.98, 5.77)	0.055
CHF (Present vs. Not present)	6.49 (0.52,81.08)	0.147	32.24 (0.12,8955.89)	0.226	22.29 (0.21,2366.4)	0.192	7.7 (0.6,99.46)	0.118
Active Smoker (Yes vs. No)	0.45 (0.12,1.64)	0.224	0.53 (0.14,2.06)	0.362	0.51 (0.13,1.98)	0.332	0.48 (0.13,1.75)	0.266
Bed Partner (Yes vs. No)	1.59 (0.68, 3.71)	0.283	1.47 (0.6, 3.56)	0.397	1.61 (0.68, 3.85)	0.281	1.51 (0.64, 3.56)	0.344
Compliance in 1 and 3 months (Yes vs. No)	12.49 (5.37, 29.03)	< 0.001	12.61 (5.18, 30.67)	< 0.001	13.29 (5.43, 32.55)	< 0.001	11.44 (4.86, 26.92)	< 0.001
L95/P95 (Continuous)			0.61 (0.43, 0.86)	0.005				
Lm/Pm (Continuous)					0.32 (0.14,0.72)	0.006		
delta AHI (Continuous)							1.07 (0.95, 1.21)	0.252

## Results

### Screening and inclusion

**Fig. 1:**
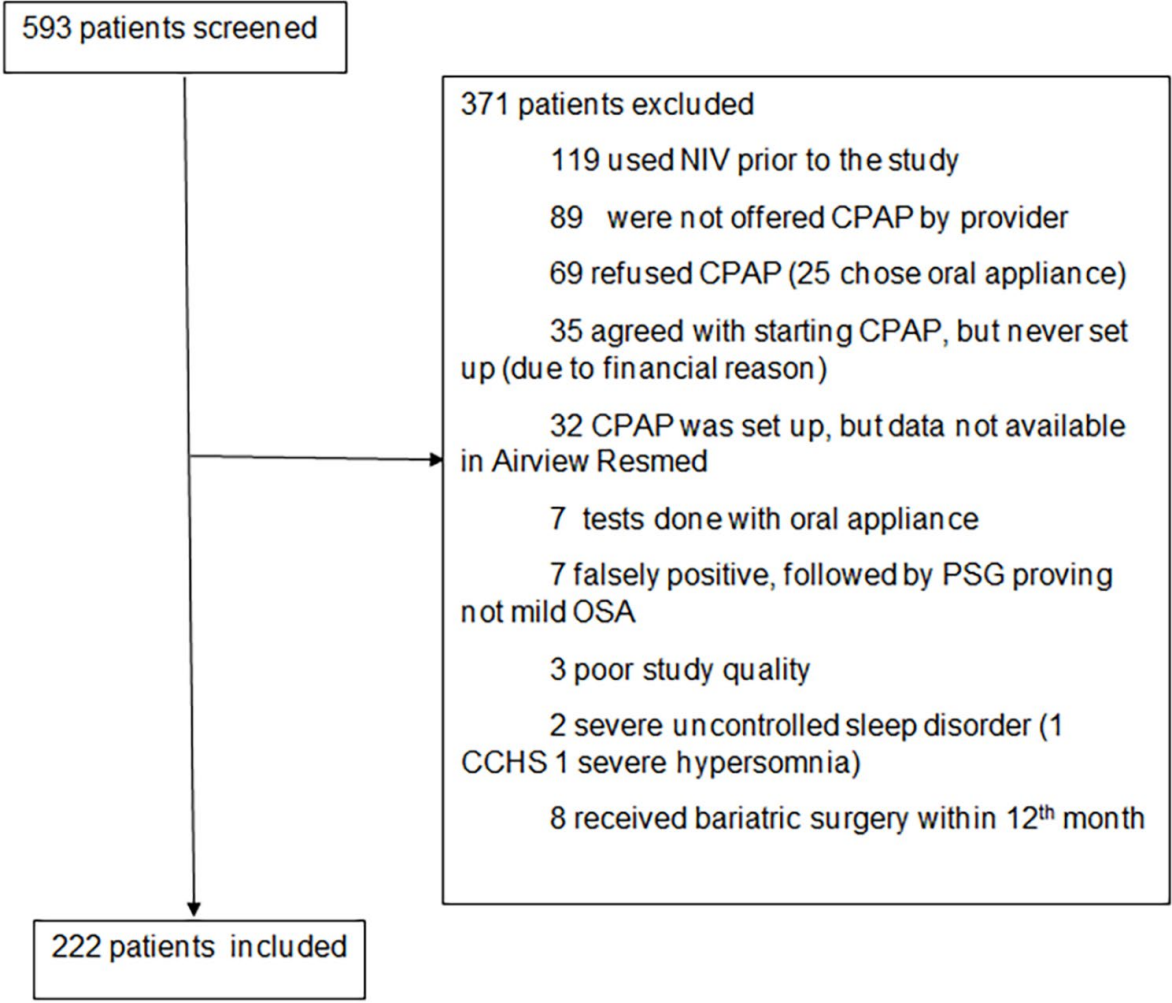
Inclusion and exclusion of the patients

We screened a total of 593 patients who were diagnosed with mild OSA by HSAT. Of these 222 were included and 371 excluded (Fig. 1).

There were 2 patients who were excluded due to severe sleep disorders other than mild OSA. One patient was found to have Congenital Central Hypoventilation Syndrome (CCHS) and the second patient was diagnosed with primary hypersomnia. The CCHS case was diagnosed through polysomnography, arterial blood gas, and eventually confirmed by genetic panel testing. The primary hypersomnia case was diagnosed through polysomnography and multiple sleep latency testing proving central hypersomnolence, and after excluding all other sleep disorders such as narcolepsy.

### Clinical characteristics

Of the 222 included patients, 57 (25.7%) were adherent and 165 (74.3%) non-adherent at the 12th month (Table 1). We found that older age, lower BMI, being a non-smoker, having a bed partner, DM, CHF, having been evaluated by the private consulting attending physician, having commercial insurance, and absence of a diagnosis of depression were all associated with a higher 12th month adherence. Compliance at any of the earlier pre-specified times (1st, 3rd, 6th and 9th month) was also associated with adherence at the 12th month (P < 0.1). Conversely, AHI, gender, race and ethnicity, prescription of auto PAP versus fixed pressure, HTN, stroke, anxiety, AF, CAD, PHT, AR, and ESS before and post treatment were not predictive of adherence at the 12th month.

### Attending physicians, insurance types and adherence at 12th month

Physician providers and insurance types were associated with differences in adherence at 12th month. There were 3 consulting attending physicians (T, K and W) who evaluated the patients in one of two clinical settings (either a community-based private office or a hospital-based clinic). Using the Fisher exact test and the adjusted p-values by false detection rate, we found that there was a significant difference in 12th month adherence between attending T and K and between T and W (Table S1). There was no significant difference between K and W, both of whom practiced in the hospital-based clinic. T practiced in the community-based private office.

Three different types of insurance categories were defined: Commercial insurance (V), Medicare(C), and Medicaid (D). We analyzed the insurance type difference between the patients of these 3 attending physicians using Fisher exact test and then adjusted the p-values by false detection rate. There was a significant difference in insurance type between T and K, and between T and W, but no difference between K and W (Table S2, S3).

Clinical characteristics between physician groups were compared (Table S4). To account for differences in physicians’ (T, K&W) clinical approach and patient population between practice settings, we introduced propensity scores. The following features were matched (1:1, nearest): race and ethnicity, BMI, smoking status, presence of a bed partner, pre-treatment ESS, and post-treatment ESS. After propensity score matching, none of the clinical characteristics between groups T and K&W remained significant (Table S5). There was also no significant difference of adherence at the 12th month between the physicians (P > 0.05).

### CPAP usage and adherence at the 12th month

Data regarding CPAP usage at 12 months were available for 207 patients. 95th percentile pressure (P95), median pressure (Pm), mask leak at the 95% percentile (L95) and median level (Lm), residual AHI and delta AHI (pretreatment AHI minus residual AHI) were analyzed using univariate analysis (Table 2) and multivariate analysis (Table 3). We found that lower pressures (P95 and Pm), lower mask leak (L95, Lm, L95/P95 and Lm/Pm), and larger delta AHI were associated with higher 12th month adherence (P < 0.1).

## Discussion

CPAP is the most commonly prescribed therapy for OSA and continues to be considered the gold standard. CPAP treatment was found to be associated with decreased cardiovascular disease risk in moderate to severe OSA [8]. Adherence with CPAP has been reported to range between 30 and 60% in previous studies [5]. Our study showed that amongst a heterogeneous population of patients with mild OSA, who agreed to CPAP treatment after being diagnosed by HSAT, only 25% were adherent at 12 months. This low adherence to CPAP therapy suggests that CPAP has a limited role in the long-term management strategy of most patients with mild OSA.

According to the AASM, treatment of mild OSA is optional and is recommended for the management of symptoms attributable to sleep apnea (poor sleep quality, daytime sleepiness, fatigue, and headache) as well as for patients with significant co-morbid illness. However, the effectiveness of CPAP treatment for many patients with mild OSA is questionable based on published data. In an 8 week, randomized placebo-controlled crossover trial, Engleman et al. reported that of the 34 patients (mean AHI 10 ± 3) enrolled, 20 patients (58.8%) preferred not to use CPAP due to inconvenience [9]. This is despite modest improvements reported in cognitive testing, depression scores, and quality of life scores after CPAP therapy. Mean use of CPAP was low at 2.8 h per night, illustrating the overall limited acceptance of CPAP in this trial [9]. In another 8 week placebo controlled study by Barnes et al., 42 patients with mild to moderate OSA received either CPAP or oral placebo [10]. Mean CPAP use was 3.53 h per night. There were no differences in Epworth Sleepiness Score, mean sleep latency or quality of life scores between groups at the end of the study [10].

These findings may reflect the observation that quality of life scores and measures of sleepiness are similar between those with mild OSA and those without OSA. In the Apnea Positive Pressure Long term Efficacy Study (APPLES), a 6 month multi-centered randomized controlled study on the impact of CPAP on neurocognitive function in OSA, there were no differences in symptoms between mild OSA and no OSA based on Epworth and Stanford Sleepiness Scales and the Sleep Apnea Quality of Life Index (SAQLI) [11]. At 6 months, CPAP had no impact on SAQLI compared to the sham group in subjects with mild OSA [11].

This is not to suggest CPAP has absolutely no role in the long-term management of mild OSA. Indeed, our study showed that one out of four patients remained adherent with CPAP at 1 year. Published data support the observation that a subset of patients with mild OSA experience benefit from CPAP therapy, particularly those with symptoms of sleepiness. In the CATNAP study, Weaver et al. reported the effects of CPAP compared to sham CPAP in subjects with mild to moderate OSA with symptoms of excessive sleepiness (ESS > 10) [12]. This study found that CPAP improved symptoms of sleepiness compared to placebo [12]. In the MERGE study, Wimms et al. reported that subjects with mild OSA treated with CPAP had a significant improvement in quality of life based on the vitality scale of the Short Form-36 questionnaire [13]. These studies suggest that symptoms of mild OSA vary from individual to individual, and treatment may be warranted in certain cases. In addition to symptom control, some studies suggest there may be some modest cardiovascular risk associated with mild OSA, such as incident hypertension in the Wisconsin Sleep Cohort, which may provide additional rationale for treatment [14].

Our study sought to describe the characteristics predictive of long-term adherence to CPAP in those with mild OSA. Older age, lower BMI, non-smoker status, presence of a bed partner, comorbid illnesses of DM and CHF, and lack of depression were all associated with higher 12th month adherence. Lower CPAP pressures, lower mask leak, and higher reduction in AHI also were associated with successful long-term CPAP use. These findings are consistent with results from prior studies [6].

We also found that differences in adherence at the 12th month were apparent early on, during the 1st and 3rd month. Out of the 57 subjects who were adherent to CPAP at the 12th month, 67% were adherent to CPAP during the 1st month and 75% were adherent during the 3rd month. In contrast, of the 165 subjects who were non-adherent to CPAP during the 12th month, only 17% were adherent to CPAP during the 3rd month. Prior studies have also found that early acceptance of CPAP predicted higher long-term adherence [15–17]. Our findings support using adherence during the initial 3 months of CPAP therapy as a predictor of long-term CPAP use in clinical practice.

It is important to note that our findings suggest CPAP compliance can differ from one patient population to another. Jacobson et al., published in 2017, evaluated long term CPAP compliance in patients who were diagnosed by home sleep testing, similar to our study [18]. Over an observation period of approximately 3 years, 54.5% of patients with mild OSA were compliant with CPAP, which is significantly higher than what we found in our study. However, our analysis included a US-based urban population, whereas the Jacobson study took place in Denmark. The differences in our findings may be related to US-specific cultural, socioeconomic, and health-care system variables that likely influence CPAP compliance. Our findings are therefore relevant to the US population, a large portion of which is urban.

The strengths of our study are the following. Our data set comes from a large community hospital-based sleep center and community-based outpatient practice. Our patient population sample represents a broad and mixed community. We included patients exclusively diagnosed using HSAT. The use of HSAT will likely continue to grow as the preferred diagnostic testing method given the cost considerations of traditional sleep testing [19]. Therefore, our findings are broadly applicable to many modern hospital-based and community sleep medicine practices in the US.

The weaknesses of our study include the limitations inherent in its retrospective design. Our study does not fully account for certain factors that may contribute to long term acceptance and adherence to CPAP therapy. These factors include adverse physical side effects from CPAP use (mask discomfort, skin irritation, feelings of claustrophobia and suffocation), financial and insurance coverage constraints, and psychological and personality variables of the individual patients. There are also variables such as use of nasal versus oronasal mask, use of heated humidifier which have been showed to affect adherence [18], but were unfortunately not well documented in the EMR and therefore was not included in our analysis. There may have been differences in how “aggressive” the various treating physicians were when offering CPAP for mild OSA that could have influenced CPAP adherence. Indeed, there were differences in CPAP adherence found between the treating physicians, although the differences were not significant after propensity score matching. It is likely that patient-related variables account for the observed differences in CPAP adherence rather than variations in physician approach to care.

In addition, using AHI as the sole criteria for categorizing severity of OSA is problematic. It is well known that HSAT results may differ from results of in-lab polysomnography, and both are susceptible to night-to-night variability [20]. It seems probable that some of our subjects who were categorized as having mild OSA (and therefore included in our study) would have been found to have a higher burden of sleep apnea if they had undergone in-lab sleep testing. Furthermore, the acceptable definition of an obstructive hypopnea differs between Medicare and other insurance carriers, which creates differences on how studies are scored and therefore how severity of OSA is determined.

Given that many practicing sleep clinicians continue to use AHI on HSAT as the primary determinant of disease severity, we felt that defining mild OSA as an AHI of 5–15 events per hour, regardless of the limitations, reflects real world clinical practice. The purpose of our study was to provide insight into how adherent these patients are to CPAP therapy over the course of 12 months. Our finding that only 25% of patients used CPAP long term are consistent with the suggestion that AHI is an imperfect measure of disease severity, and the value of CPAP treatment from one individual may be significantly different from another despite sharing the diagnosis of mild disease [21].

## Conclusions

In conclusion, in patients with mild OSA diagnosed by HSAT, long term adherence to CPAP therapy is low. Early acceptance of CPAP therapy in the first 3 months predicts long term adherence to therapy. Our findings support the use of a limited 3-month trial of CPAP therapy for symptomatic or at risk patients with mild OSA. Early adoption of other therapies (mandibular advancement devices, positional therapy, cognitive and behavioral treatment, pharmacologic interventions, and/or surgery) or adherence interventions should be strongly considered in patients who are non-adherent after an initial time limited trial. Further studies are needed to help clarify which long-term management strategies are most effective for patients with mild OSA who are non-adherent with CPAP.

### Electronic supplementary material

Below is the link to the electronic supplementary material.

Supplementary Material 1

Supplementary Material 2

## Data Availability

The datasets supporting the conclusions of this article are included within the article and its additional files.

## Abbreviations

AF: Atrial fibrillation
AHI: Apnea-hypopnea index
AR: Allergic rhinitis
AUC: Area under the curve
BMI: Body mass index
CAD: Coronary artery disease
CHF: Congestive heart failure
CPAP: Continuous Positive Airway Pressure
DM: Diabetes Mellitus
ESS: Epworth Sleepiness Scores
HTN: Hypertension
HSAT: Home sleep apnea testing
OSA: Obstructive sleep apnea
PHT: Pulmonary hypertension
