# Supplementary material for: Long term adherence to continuous positive Airway pressure in mild obstructive sleep apnea
Source: BMC Pulm Med. 2023 Sep 1;23:320. doi: 10.1186/s12890-023-02612-3 (PMC10472589; doi:10.1186/s12890-023-02612-3)
Supplement: Supplementary file 1 — Supplementary Material 1 [file 12890_2023_2612_MOESM1_ESM.docx]

Table S1: 12^th^ month compliance difference between three attending physicians

| Comparison | P-value(Fisher) | P-value( adj. Fisher) |
| --- | --- | --- |
| T:K | 0.0355 | 0.0532 |
| T:W | 0.0145 | 0.0435 |
| K:W | 1.0000 | 1.0000 |

Table S2: Insurance types (V, C, D) difference between different attending physicians

|  | V | C | D |
| --- | --- | --- | --- |
| T | 57 | 5 | 13 |
| K | 22 | 4 | 29 |
| W | 32 | 9 | 51 |
| p value | 4.363e-07 | | |

Table S3: Insurance comparison between attending physicians

| Comparison | p-value(Fisher) | p-value(adj. Fisher) |
| --- | --- | --- |
| T:K | 4.63e-05 | 6.95e-05 |
| T:W | 1.63e-07 | 4.89e-07 |
| K:W | 0.8166 | 0.8166 |

Table S4: comparison of patients’ clinical characteristics between two groups of attending physicians

| Clinical Characteristics | | K&W | T | P-value |
| --- | --- | --- | --- | --- |
|  |  | N=147 | N=75 |  |
| Age in years | Mean | 48.5 | 51.7 | 0.05132 |
|  | Range | (20,74) | (27,79) |  |
| Gender | Female | 90 | 40 | 0.3134 |
|  | Male | 57 | 35 |  |
| Race and Ethnicity | White | 37 | 47 | 2.221e-07 |
|  | African | 39 | 13 |  |
|  | Asian | 1 | 1 |  |
|  | Hispanic | 70 | 14 |  |
| BMI | Mean | 36.5 | 32.5 | 7.033e-05 |
|  | Range | (23,67) | (21,57) |  |
| Settings | auto PAP | 135 | 72 | 0.3964 |
|  | Fixed pressure | 12 | 3 |  |
| HTN | No | 64 | 37 | 0.4766 |
|  | Yes | 83 | 38 |  |
| Depression | No | 113 | 60 | 0.7325 |
|  | Yes | 34 | 15 |  |
| Anxiety | No | 112 | 62 | 0.3043 |
|  | Yes | 35 | 13 |  |
| Stroke | No | 145 | 75 | 0.5506 |
|  | Yes | 2 | 0 |  |
| AF | No | 143 | 70 | 0.1696 |
|  | Yes | 4 | 5 |  |
| DM | No | 103 | 57 | 0.4295 |
|  | Yes | 44 | 18 |  |
| CAD | No | 137 | 74 | 0.1037 |
|  | Yes | 10 | 1 |  |
| CHF | No | 144 | 72 | 0.4085 |
|  | Yes | 3 | 3 |  |
| PHT | No | 145 | 74 | 1 |
|  | Yes | 2 | 1 |  |
| AR | No | 126 | 58 | 0.1334 |
|  | Yes | 21 | 17 |  |
| AHI | Median | 8.9 | 9.6 | 0.4598 |
|  | Range | (5,14.7) | (5.1,14.9) |  |
| Active smoker | No | 120 | 70 | 0.02495 |
|  | Yes | 27 | 5 |  |
| Bed Partner | No | 63 | 19 | 0.01237 |
|  | Yes | 84 | 56 |  |
| ESS before therapy | Median | 10 | 8 | 0.03672 |
|  | Range | (0,24) | (0,21) |  |
| ESS after treatment | Median | 5 | 4 | 0.01197 |
|  | Range | (1,14) | (0,14) |  |
| Compliance at 12th month  (C12) | No | 118 | 47 | 0.005777 |
|  | Yes | 29 | 28 |  |

Table S5: Comparison of propensity matched patients between two groups of attending physicians

| Characteristics | | K&W | T | P-value |
| --- | --- | --- | --- | --- |
|  |  | N=75 | N=75 |  |
| Age in years | Mean | 50.6 | 51.7 | 0.5598 |
|  | Range | (20,74) | (27,79) |  |
| Gender | Female | 37 | 40 | 0.744 |
|  | Male | 38 | 35 |  |
| Race and Ethnicity | NW | 36 | 47 | 0.2572 |
|  | AA | 16 | 13 |  |
|  | AS | 1 | 1 |  |
|  | H | 22 | 14 |  |
| BMI | Mean | 33.4 | 32.5 | 0.3276 |
|  | Range | (23,52) | (21,57) |  |
| Settings | Auto PAP | 70 | 72 | 0.719 |
|  | Fixed Pressure | 5 | 3 |  |
| HTN | No | 34 | 37 | 0.7438 |
|  | Yes | 41 | 38 |  |
| Depression | No | 55 | 60 | 0.4403 |
|  | Yes | 20 | 15 |  |
| Anxiety | No | 59 | 62 | 0.6797 |
|  | Yes | 16 | 13 |  |
| Stroke | No | 74 | 75 | 1 |
|  | Yes | 1 | 0 |  |
| AF | No | 73 | 70 | 0.4419 |
|  | Yes | 2 | 5 |  |
| DM | No | 57 | 57 | 1 |
|  | Yes | 18 | 18 |  |
| CAD | No | 68 | 74 | 0.06305 |
|  | Yes | 7 | 1 |  |
| CHF | No | 73 | 72 | 1 |
|  | Yes | 2 | 3 |  |
| PHT | No | 73 | 74 | 1 |
|  | Yes | 2 | 1 |  |
| AR | No | 63 | 58 | 0.4086 |
|  | Yes | 12 | 17 |  |
| AHI | Median | 9 | 9.6 | 0.8494 |
|  | Range | (5.1,14.7) | (5.1,14.9) |  |
| Active smoker | No | 65 | 70 | 0.2761 |
|  | Yes | 10 | 5 |  |
| Bed Partner | No | 22 | 19 | 0.7143 |
|  | Yes | 53 | 56 |  |
| ESS before treatment | Median | 8 | 8 | 0.9489 |
|  | Range | (0,24) | (0,21) |  |
| ESS after treatment | Median | 4 | 4 | 0.3259 |
|  | Range | (1,14) | (0,14) |  |
| Compliance at 12th month (C12) | No | 58 | 47 | 0.07418 |
|  | Yes | 17 | 28 |  |
